# Signalling pathways involved in urotensin II induced ventricular myocyte hypertrophy

**DOI:** 10.1371/journal.pone.0313119

**Published:** 2025-01-16

**Authors:** Hadeel S. Al Ali, Glenn C. Rodrigo, David G. Lambert

**Affiliations:** 1 Department of Cardiovascular Sciences, Clinical Sciences Wing, Glenfield Hospital, University of Leicester, Leicester, United Kingdom; 2 Department of Physiology, Al-Zahraa College of Medicine, University of Basrah, Basrah, Iraq; 3 Department of Cardiovascular Sciences, Anaesthesia, Critical Care and Pain Management, University of Leicester, Leicester, United Kingdom; Nanjing University, CHINA

## Abstract

Sustained pathologic myocardial hypertrophy can result in heart failure(HF); a significant health issue affecting a large section of the population worldwide. In HF there is a marked elevation in circulating levels of the peptide urotensin II(UII) but it is unclear whether this is a result of hypertrophy or whether the high levels contribute to the development of hypertrophy. The aim of this study is to investigate a role of UII and its receptor UT in the development of cardiac hypertrophy and the signalling molecules involved. Ventricular myocytes isolated from adult rat hearts were treated with 200nM UII for 48hours and hypertrophy was quantified from measurements of length/width (L/W) ratio. UII resulted in a change in L/W ratio from 4.53±0.10 to 3.99±0.06; (p<0.0001) after 48hours. The response is reversed by the UT-antagonist SB657510 (1μM). UT receptor activation by UII resulted in the activation of ERK1/2, p38 and CaMKII signalling pathways measured by Western blotting; these are involved in the induction of hypertrophy. JNK was not involved. Moreover, ERK1/2, P38 and CaMKII inhibitors completely blocked UII-induced hypertrophy. Sarcoplasmic reticulum (SR) Ca^2+^-leak was investigated in isolated myocytes. There was no significant increase in SR Ca^2+^-leak. Our results suggest that activation of MAPK and CaMKII signalling pathways are involved in the hypertrophic response to UII. Collectively our data suggest that increased circulating UII may contribute to the development of left ventricular hypertrophy and pharmacological inhibition of the UII/UT receptor system may prove beneficial in reducing adverse remodeling and alleviating contractile dysfunction in heart disease.

## Introduction

Cardiac hypertrophy has been identified as one of the greatest independent risk factors for cardiac morbidity [1]. Much effort has been concentrated around characterizing the intracellular signalling pathways involved in, and the neurohormonal stimuli that are associated with ventricular myocyte hypertrophy. Ventricular myocyte hypertrophy develops as an adaptive response when cells are stimulated with a wide array of growth stimuli. Sustained hypertrophy is often significantly associated with an increase in the risk of progression to heart failure. Cardiovascular regulating factors that participate in cardiac hypertrophy; exemplified by angiotensin II (Ang II), endothelin-1 (ET-1) and catecholamines (α-adrenergic), bind to G protein coupled receptors (GPCR) [2]. The mitogen-activated protein kinase (MAPK) family has been reported to be important for induction of cardiac hypertrophy and has been widely investigated [3–12], including extracellular-regulated kinases (ERKs), p38 mitogen-activated protein kinases and c-Jun N-terminal kinases (JNK)] [3, 13, 14]. ERK1/2 is involved in many signal transduction pathways that control a variety of cellular physiological processes such as differentiation, proliferation, growth, stress responses, motility and apoptosis [15–18]. The p38 MAPK has also a prominent role in cellular regulation [19–21], especially inflammation [22, 23].

Urotensin II (UII) is a neurohormonal peptide; both peptide and its receptor (UT) are ubiquitously expressed in cardiac tissue of patients with end stage heart failure where it is mainly increased in myocytes but to a lesser extent also in endothelial cells and vascular smooth muscle cells [24, 25]. Whilst levels of circulating UII are associated with hypertrophic heart failure, its link to ventricular hypertrophy is not clear. Similarly, whether UII contributes to contractile dysfunction associated with heart failure is also not clear. Ng and colleagues reported circulating UII levels in healthy volunteers were 9.16pg/ml and these increased to 30.6pg/ml in patients with heart failure [26]. In another study, Richards and colleagues reported UII levels of 5.4pg/ml in patients with chronic heart failure compared to a healthy group at 2.6pg/ml [27]. This elevation in UII in heart failure can depress left ventricular contractility [24]. Furthermore, the relationship between ejection fraction and UII in patients with heart failure has been investigated; there is an inverse relationship [28].

*In vitro* and *in vivo* experimental studies have been undertaken to identify the role of UII in cardiac and vascular remodeling which contributes to the progression of cardiovascular diseases [14, 29–32]. These studies generally have included monitoring the effect of UII either on morphological changes and protein synthesis via treatment of cells in culture with UII or determination of structural and functional changes to the cardiovascular system following administration of the peptide directly to animals.

The hypothesis of this study is that UII promotes strong phenotypic changes and as plasma levels of UII are elevated in heart failure, they may be involved in the development of pathological cardiac hypertrophy. The aim of this study was to determine involvement of the UII/UT system (UII and its receptor) in hypertrophy of ventricular myocytes and the cellular mechanism responsible.

## Materials and methods

### Isolation of ventricular myocytes and cell culture

This study was approved by University of Leicester Animal Ethics committee (AWERB_2024_214 dated 25th Feb 2024). Adult male Wistar rats (body weight 200-300g) were bred and held in specific environmentally controlled conditions in the Pre-clinical Research Facility, University of Leicester. Rats were euthanized by a sharp blow to the head followed by cervical dislocation (in compliance with schedule 1 of the Animals, Scientific Procedures Act, 1986).

Adult rat ventricular myocytes (ARVMs) were isolated by enzymatic digestion, using a method previously described, resulting in 70–80% viable rod shaped cells [33]. Ventricular myocytes were cultured in six-well plates. 2ml of cellular suspension was added to each well and the cells were incubated for up to 48 hours at 37°C in an atmosphere of 5% CO_2_.

### Induction and quantification of hypertrophy in ARVMs

Cultured ARVMs were treated with 200nM UII (hUII was a gift from Professor R Guerrini, University Ferrara, Italy) or rUII (Sigma-Aldrich) to induce hypertrophy. 10μM phenylephrine (PHE) was used as a positive control [34]. Untreated ARVMs were used as further controls and media was replaced after 24 hours. The morphology and dimensions of single ventricular myocytes were quantified with ‘straight line ImageJ software’ from light microscope images after treating cells with UII or phenylephrine; length width ratio was calculated. This decreases with pathophysiological hypertrophy, as the width increases. Measurements were made blind.

### Pharmacological dissection of the hypertrophic response to UII

To confirm UT receptor activation cultured ARVMs were treated with 1μM SB657510, a UT receptor antagonist, for 15 minutes prior to stimulation with 200nM UII. Signalling profiles were probed with appropriate pharmacological inhibitors. These were PD184352 (ERK1/2 inhibitor, prepared in DMSO and applied at 5μM) [35], SB202190 (p38 inhibitor, prepared in DMSO and applied at 10μM) [36] or KN-93 (CaMKII inhibitor, prepared in Milli-Q water and applied at 5μM) [37] and were preincubated for 30 minutes prior to stimulation with 200nM UII. (PD184352 and KN-93 inhibitors were purchased from Sigma-Aldrich while SB202190 was purchased from Synkinase).

### Cell lysis and Western blot analysis

Isolated ARVMs were cultured in 6 well plates using media 199 plus HEPES. After 24 hours, the cultured myocytes were stimulated with 200nM UII for different durations to define the time course for signalling pathway activation. Cells were centrifuged at 1,000g for 1 minute using a mini-centrifuge (Eppendorf). The cells were lysed using RIPA buffer (100μL) and stored at -20°C until required. To identify target proteins, 40μL of denatured protein alongside 3μL of blue protein ladder and biotin ladder were run through 10% sodium dodecyl sulphate-polyacrylamide gel electrophoresis (SDS-PAGE) mini-gels. Immediately after electrophoresis of the protein samples, the gels were transferred onto a nitrocellulose membrane (Fisher Scientific) via an overnight wet transfer at 30V at room temperature. Then, the membranes were incubated overnight with the primary antibody of interest, phosphorylated-ERK1/2 (p-ERK1/2, Cell Signalling; 1μl in 6ml Tris-buffered saline with tween 20 (TBS-T), phosphorylated-p38 (p-p38, Cell Signalling; 1μl in 3ml TBS-T), phosphorylated-JNK (p-JNK, Cell Signalling; 1μl in 3ml TBS-T) and phosphorylated CaMKII (p-CaMKII, Abcam; 1μl in 3ml TBS-T) at 4°C with gentle agitation. Following incubation the membranes were washed for 30 minutes with TBS-T to remove unbound antibody, the washing process being repeated six times for 5 minutes. The nitrocellulose membranes were then incubated in the appropriate secondary antibody, anti-rabbit IgG with horseradish peroxidase (Cell Signalling) for 1 hr diluted in TBS-T (10μL in 10ml) containing 5% skimmed milk powder and 10μL of anti-biotin secondary antibody. Incubation was at room temperature with gentle agitation. Membranes were subsequently washed with TBS-T six times for 30 minutes. Density of immunoreactive bands was visualized and detected using a Biorad ChemiDoc^™^ MP imager (BIO-Rad). The membranes were stripped and reprobed with appropriate new antibody specific to the total protein of interest (Cell Signalling; 1μl in 3ml TBS-T). Levels of total protein were quantified and used for normalisation of the phosphorylated target protein. Uncropped blots are shown in S1 Raw images.

### UT expression

Assessment of UT receptor mRNA expression in ARVMs was performed by Reverse transcription polymerase chain reaction (RT-PCR) assay as in reference [38] using kits according to manufacturer’s instructions. As this was presence/absence analysis Glyceraldehyde 3-phosphate dehydrogenase (GAPDH) was used as a single reference gene. Cycle threshold (C_t_) is presented for GAPDH and UT receptor with the difference ΔC_t_ presented to describe expression. There was no amplification in non-template controls.

### Measurement of intracellular calcium

Intracellular Ca^2+^ was measured in single cultured ventricular myocytes loaded with Fura-2AM (acetoxymethyl ester) [39] with detail as in [40].

Calibration of Fura-2 fluorescence was carried out using the method previously described by Hunt and Lambert, 2013 [41]. A Ca-dissociation constant (K_d_) of 285nM was used [42]. R_min_ (0.4) and R_max_ (4.96) was determined in myocytes loaded with 2mM BAPTA-AM to buffer Ca^2+^ to very low levels, or in myocytes mechanically disrupted with a microelectrode; the ratio of bound to unbound Ca^2+^ was determined at 380nm (F_380max_/F_380min_) from 41 cells. [Ca]_i_ in nM was calculated using the following equation:

$${\left[\text{Ca}\right]}_{\text{i}}={\text{K}}_{\text{d}}(\text{R}-{\text{R}}_{\text{min}}/{\text{R}}_{\text{max}}-\text{R})\times ({\text{F}}_{380\;\text{max}}/F_{380\;\text{min}})$$

To determine sarcoplasmic reticulum Ca-leak, cells were loaded with 10μM Fluo-3 for 40 minutes and excited at a wavelength of 506nm. Fluo-3 was used, as it has a very low signal-to-noise ratio, and so facilitates measurement of small changes in SR Ca-leak. The diastolic calcium concentration [Ca]_d_ was measured from myocytes at the beginning of experiment using Fura-2. The diastolic fluorescence (F_0_) was determined using Fluo-3, between at least 6 electrically-induced Ca-transients and the mean used. K_d(Ca)_ represents the affinity of Fluo-3 for calcium and was measured to be 1100nM. Subsequently, Fluo-3 fluorescence was calibrated utilizing a pseudo ratio according to the equation below:

$${\left[\text{Ca}\right]}_{\text{i}}=\text{F}/{\text{F}}_{0}\left({\text{K}}_{\text{d}(\text{Ca})}\right)/\left(\left({\text{K}}_{\text{d}\left(\text{Ca}\right)}/{\left[\text{Ca}\right]}_{\text{d}}\right)-\text{F}/{\text{F}}_{0}+1\right)$$

### Sarcoplasmic reticulum Ca^2+^-leak

**Fig 1:**
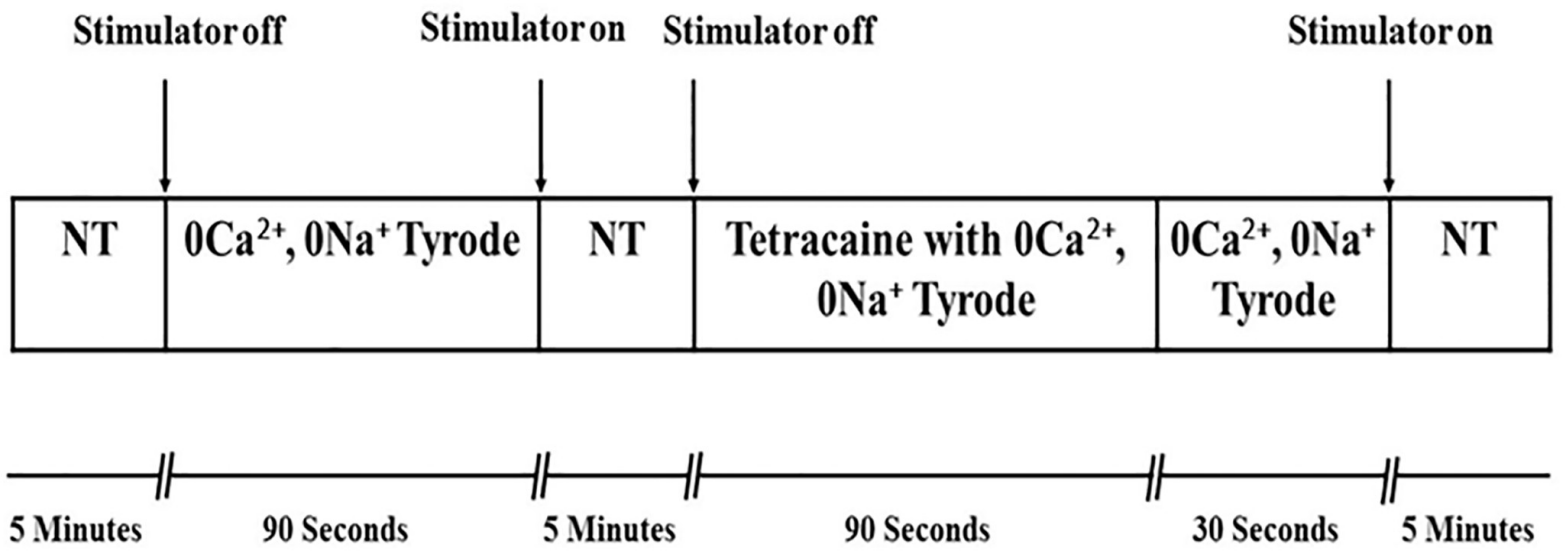
Schematic diagram of the sarcoplasmic reticulum Ca^2+^-leak experiment. Ventricular myocytes loaded with Fluo-3 were used to determine SR Ca^2+^-leak, using tetracaine (1mM) in EGTA solution to block Ca^2+^-leak through ryanodine receptors (RyR2).

The calcium fluorescent indicator Fluo-3 was used to measure resting [Ca^2+^] in the presence and absence of tetracaine. Fluo-3 has maximum excitation wavelength at 506nm and emission wavelength at 525nm [43, 44]. Cells were perfused in normal Tyrode and stimulated at 1Hz until steady-state. Ca^2+^-transients were recorded and then the stimulator switched off and cells were superfused with Ca^2+^ and Na^+^-free Tyrode (10mmol Ethylene glycol-bis(β-aminoethyl ether)-N,N,N’,N’-tetraacetic acid (EGTA)), to determine diastolic [Ca^2+^]_i_ in the absence of Ca^2+^-flux across the cell membrane (L-type calcium channels and Sodium/calcium exchanger (NCX)). Following this the cells were superfused with normal Tyrode and electrically stimulated at 1Hz until the normal Ca^2+^-transient was recorded. The cells were then superfused with 1mM tetracaine in EGTA solution (0Ca^2+^, 0Na^+^) for 90 seconds (Fig 1). Tetracaine blocks ryanodine receptor channels (RyR2) and prevents Ca^2+^-leak from the sarcoplasmic reticulum and in the absence of Ca^2+^-flux across the cell membrane diastolic [Ca^2+^]_i_ declines reflecting the absence of Ca^2+^-leak though RyR2 [45].

### Statistical analysis

Data are shown as number of hearts/animals; number of cells. All data from experiments were arranged and tabulated in Excel. Results were expressed as mean ± S.E.M. Data were analyzed by one-way ANOVA with *post hoc* testing or t-test as appropriate and indicated in the figure legends using GraphPad Prism 7.0 software (Graphpad, Version 7.0, US). P-value <0.05 was considered as significant.

## Results

### UT expression in ARVMs

UT mRNA expression in ARVMs was assessed by RT-PCR using GAPDH as the reference gene. Freshly isolated ventricular myocytes from 9 adult rat hearts were used. The mean C_t_ value for GAPDH was 13.84 ± 2.20 while that for UT was 35.0 ± 1.40 giving a ΔC_t_ value of 21.18 ± 3.12.

### UII drives hypertrophy in adult ventricular myocytes in primary culture

**Fig 2:**
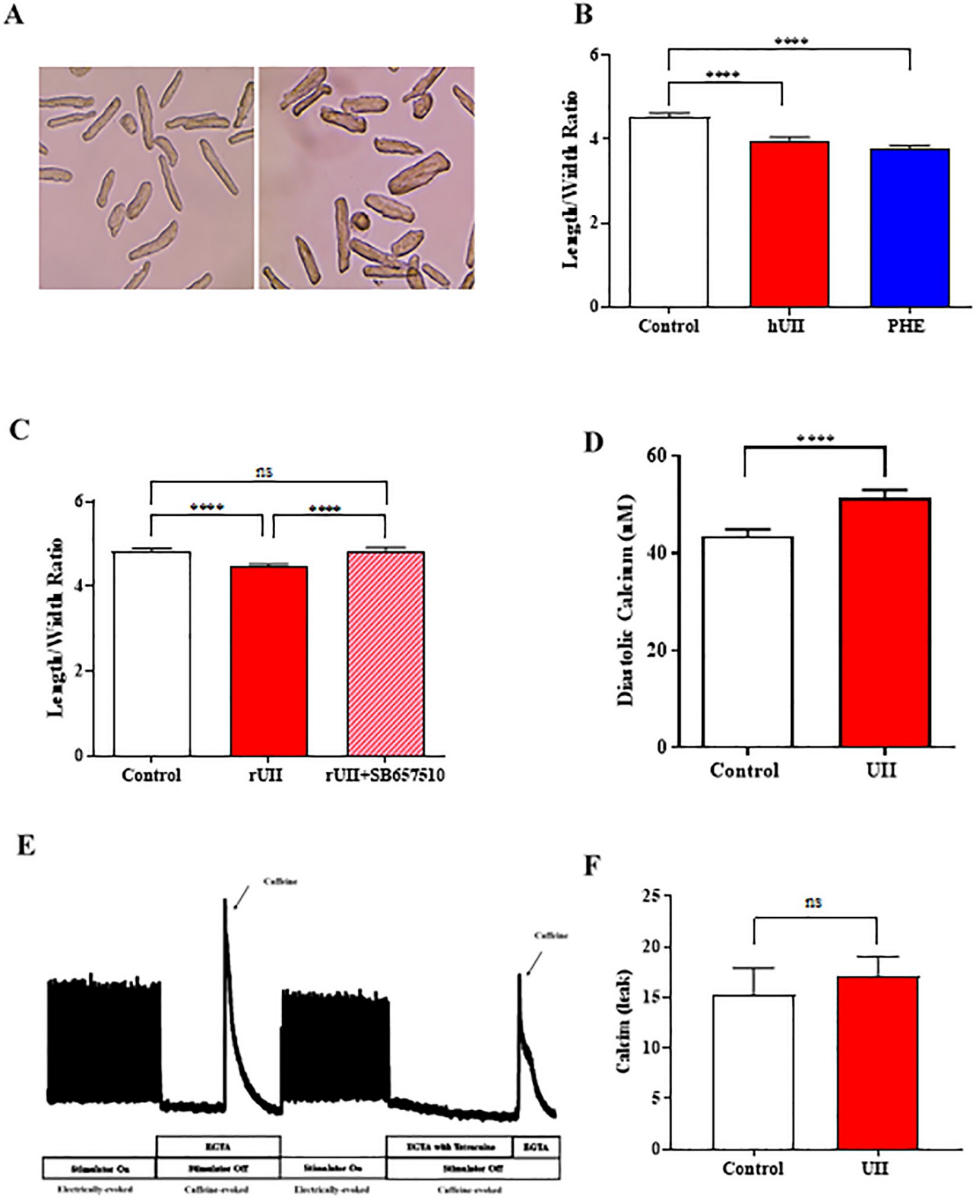
Ventricular myocyte hypertrophy and SR Ca^2+^-leak. (A) Isolated ventricular myocyte images in culture media (left). Ventricular myocytes treated with 200nM UII for 48 hours (right). (B) Length/width ratio in response to hUII and phenylephrine (PHE). Measurement of length/width ratio after 48 hours treatment with hUII (200nM) or phenylephrine (10μM). N = 6 hearts; 126, 209 & 141 cells, respectively. ****p<0.0001. One-way ANOVA followed by Sidak’s *post hoc* test. (C) Effect of UT receptor antagonist on rUII induced hypertrophy. Measurement of length/width ratio after rUII (200nM) or cells pretreated with SB657510 (1μM) before exposure to rUII for 24 hours. N = 6 hearts; 420, 519 & 441 cells, respectively. ****p<0.0001. One-way ANOVA followed by Sidak’s *post hoc* test. (D) Measurement of intracellular diastolic Ca^2+^-transient in cultured ARVMs. Diastolic [Ca^2+^]_i_ recorded from ventricular myocytes after 24 hours tissue culture in the absence (control) and presence of 200nM UII. N = 8 hearts; 94 cells. ****p<0.0001. Paired t-test. (E) An example recording of showing the protocol and [Ca^2+^]_i_ to determine SR Ca^2+^-leak. (F) Diastolic SR Ca^2+^-leak (nM) in cultured ventricular myocytes. Cells cultured for 24 hours in the absence (control) and presence of UII (200nM) for 24 hours. The difference in the diastolic [Ca^2+^]_i_ was measured in the presence and absence of 1mM tetracaine for both control and treated cells. N = 6 hearts; 19 control cells & 25 treated cells. Unpaired t-test. All results are expressed as mean ± S.E.M.

Ventricular myocytes demonstrated characteristic phenotypic changes associated with pathophysiological hypertrophy, in response to either phenylephrine or UII (Fig 2), as indicated by significant decrease in length/width ratio.

Phenylephrine caused a significant reduction in length/width ratio after 48 hours from 4.53 ± 0.10 (N = 6 hearts; 126 cells) to 3.77 ± 0.08 (N = 6 hearts; 141 cells) (p<0.0001) (Fig 2B). These data demonstrate the ability of our tissue culture system to show a significant level of hypertrophy in response to a well-documented hypertrophic stimulus [34].

Similarly, treatment of ventricular myocytes with hUII (200nM) led to a significant reduction in length/width ratio after incubation for 48 hours from 4.53 ± 0.10 (N = 6 hearts; 126 cells) to 3.99 ± 0.06 (N = 6 hearts; 209 cells); (p<0.0001) (Fig 2B). Ventricular myocytes stimulated with hUII (200nM) became hypertrophied at a level equivalent to that produced by phenylephrine (10μM).

To show that the hypertrophic effect of UII on ventricular myocytes involved binding of UII to the UT receptor (identified by PCR), the cells were stimulated with rUII (200nM) in primary culture for 24 hours in the presence or absence of the UT receptor antagonist SB657510. Length/width ratio following incubation with rUII significantly decreased (4.48 ± 0.05, N = 6 hearts; 519) compared to control (4.83 ± 0.0.59, N = 6 hearts; 420 cells) after 24 hours (p<0.0001) (Fig 2C), these differences measured with rUII were consistent with the previous hUII data. Cultured ventricular myocytes were pretreated with 1μM SB657510 antagonist [46] for 15 minutes before addition/and incubation with rUII 200nM. The hypertrophic response of ARVMs to UII was reversed by SB657510 (4.85 ± 0.06, N = 6 hearts; 441 cells) (p<0.0001) (Fig 2C).

The ability of the selective UII receptor antagonist SB657510 to block the hypertrophic response to UII, shows the direct involvement of UII receptor.

### Effect of UII on sarcoplasmic reticulum Ca^2+^-leak

Pathological cardiac hypertrophy is associated with dysregulation of Ca^2+^ cycling and abnormalities of electrical activity [47]. Consequently, the effects of chronic exposure of ARVMs to 200nM UII for 24 hours on Ca^2+^-regulation of ventricular myocytes were investigated. Cells were placed into tissue culture in the presence of 200nM UII for 24 hours. Diastolic [Ca^2+^]_i_ increased from to 43.5 ± 1.4nM in control cells cultured in the absence of UII to 51.3 ± 1.6nM in cells cultured for 24 hours in the presence of UII (200nM) (N = 8 hearts; 94 cells, p<0.0001) (Fig 2D).

To determine whether the increase in diastolic [Ca^2+^]_i_ by UII resulted from an increased sarcoplasmic reticulum (SR) Ca^2+^-leak, tetracaine was used to block RyR2 leak whilst measuring diastolic Ca^2+^ in the absence of sarcolemmal Ca^2+^-fluxes [45], an example [Ca^2+^]_i_ trace recorded from single ARVM is shown in Fig 2E. Diastolic [Ca^2+^]_i_ increased from 15.3 ± 2.6nM in untreated ventricular myocytes cultured for 24 hours (N = 6 hearts; 19 cells) to 17.1 ± 2nM in UII treated cells cultured for 24 hours (N = 6 hearts; 25 cells) (p>0.05) (Fig 2F).

### UII activates MAPK and induces phosphorylation of CaMKII in ventricular myocytes

**Fig 3:**
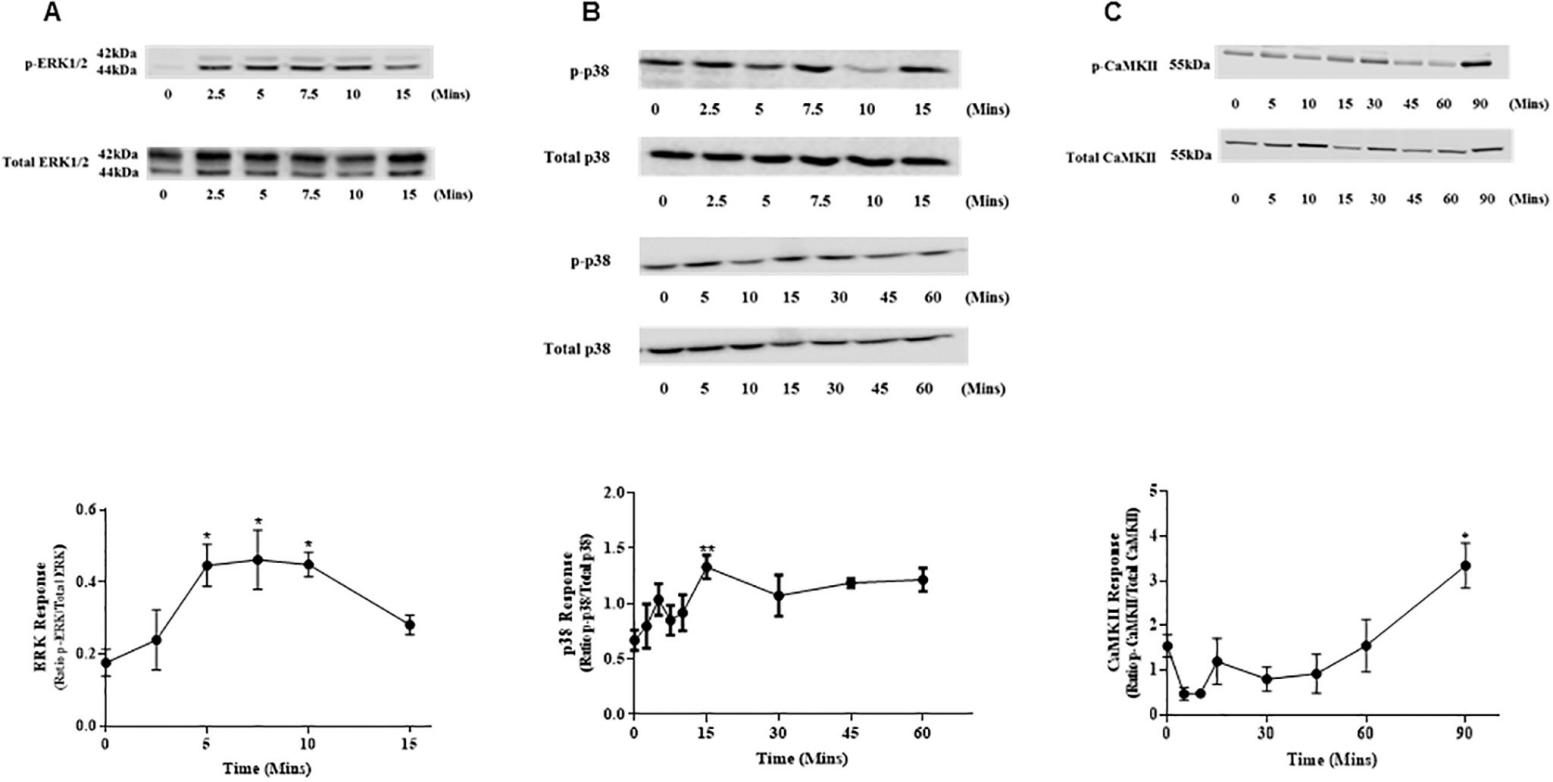
Activation of MAPK and CaMKII in ventricular myocytes. (A) Phosphorylation time course of ERK1/2 in ventricular myocytes by 200nM UII. Phosphorylation of ERK1/2 and total ERK was detected by Western blot analysis. N = 6 hearts. *p<0.05. (B) UII (200nM) induced p38 phosphorylation time course in ventricular myocytes. Ventricular myocyte p38 response was detected by Western blot analysis. N = 7 hearts. **p<0.01. (C) Time course for UII-induced phosphorylation of CaMKII in ventricular myocytes after treatment with 200nM UII. Phosphorylation of CaMKII and total CaMKII was detected by Western blot analysis. N = 5 hearts. *p<0.05. The results are expressed as mean ± S.E.M. One-way ANOVA followed by Dunn’s post hoc test. Uncropped blots are shown in the S1 File.

Urotensin II acting at the UT receptor increases activation of ERK1/2 signalling pathway [48]. ARVMs from 6 rat hearts were cultured for 24 hours prior to stimulation with UII (200nM). UII increased ERK phosphorylation (ratio) from basal of 0.17 ± 0.03 to 0.44 ± 0.05 (p<0.05) after 5 minutes, and reached a plateau of 0.46 ± 0.08 (p<0.05) after 7.5 minutes declining to 0.28 ± 0.02 (p<0.05) at 15mins (Fig 3A).

There is data showing coupling of UT to p38 in endothelial progenitor cells [49]; this was examined in ARVMs in a longer time course. Basal phosphorylation was 0.67 ± 0.09 and this increased after 15 minutes to 1.33 ± 0.10 (p<0.01) and remained elevated until sampling ended at 60 minutes (Fig 3B). There was no change in JNK phosphorylation over a 45min time course (S1 Raw images).

Previous studies have shown that alterations in CaMKII isoforms are associated with hypertension which induces hypertrophy and can result in heart failure [50, 51]. It is possible that these changes in CaMKII influence intracellular Ca^2+^ handling which may cause abnormal ventricular contractility [50, 52]. As we observed an increase in diastolic Ca^2+^ levels in response to chronic exposure to UII, we looked at the ability of UII to induce phosphorylation of CaMKII in ARVMs. There was a significant increase in phosphorylation from basal of 1.54 ± 0.25 to 3.34 ± 0.5 after 90 minutes (p<0.05) (Fig 3C).

**Fig 4:**
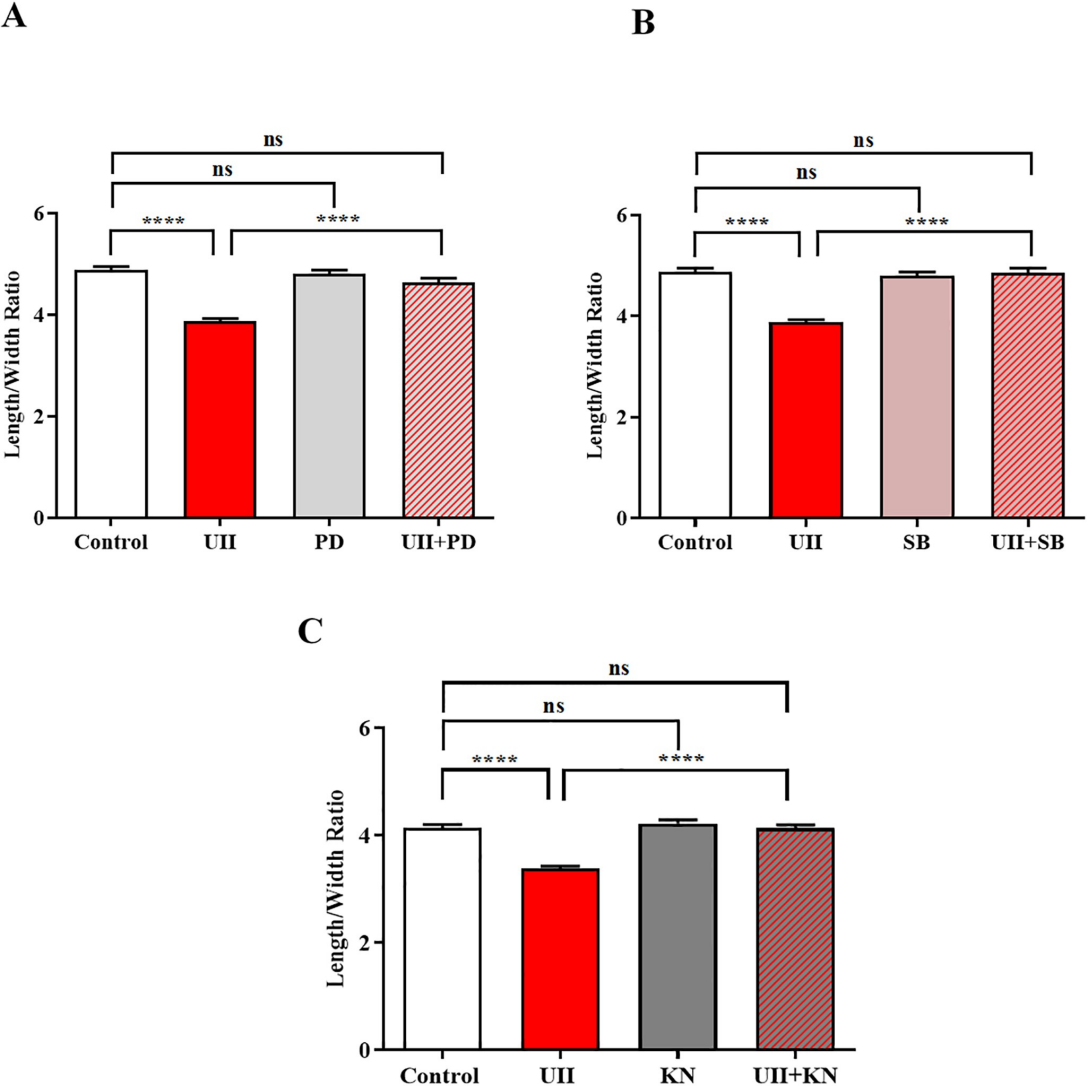
Role of MAPK and CaMKII in UII-induced myocyte hypertrophy. (A) Effect of ERK1/2 inhibitor (PD184352; PD) on UII-induced hypertrophy in cultured ventricular myocytes. PD184352 blocked the UII-induced hypertrophy after 48 hours. N = 6 hearts; 358, 420, 253 & 195 cells, respectively. ****p<0.0001. (B) Effect of p38 inhibitor (SB202190; SB) on UII-induced hypertrophy in cultured ventricular myocytes. SB202190 blocked UII-induced hypertrophy after 48 hours. N = 6 hearts; 358, 420, 278 & 212 cells, respectively. ****p<0.0001. (C) Effect of CaMKII inhibitor (KN-93; KN) on UII-induced hypertrophy in cultured ventricular myocytes. KN-93 blocked UII-induced hypertrophy after 48 hours. N = 6 hearts; 320, 386, 282 & 223 cells, respectively. ****p<0.0001. Results are expressed as mean ± S.E.M. One-way ANOVA followed by Sidak’s *post hoc* test.

Cultured ventricular myocytes were treated with 5μM of the ERK1/2 inhibitor PD184352 for 30 minutes prior to stimulation with 200nM UII in order to probe the role of ERK1/2 signalling in UII-induced hypertrophy (L/W ratio) [35]. Inhibition of ERK1/2 signalling pathway completely blocked the UII-induced hypertrophy after 48 hours from (3.87 ± 0.05, N = 6 hearts; 420 cells) in UII group to (4.64 ± 0.08, N = 6 hearts; 195 cells) in UII+PD184352 group (p<0.0001) (Fig 4A). In the case of PD184352 we also made some measurements at 24hours in the same cell batch where UII induced hypertrophy was fully reversed and the inhibitor alone was inactive.

Cultured ventricular myocytes were treated with 10μM of the p38 inhibitor SB202190 for 30 minutes prior to stimulation with 200nM UII in order to probe the role of p38 signalling in UII-induced hypertrophy [36]. Inhibition of p38 signalling pathway completely blocked UII-induced hypertrophy after 48 hours from (3.87 ± 0.05, N = 6 hearts; 420 cells) in UII group to (4.85 ± 0.08, N = 6 hearts; 212 cells) in UII+SB202190 group (p<0.0001) (Fig 4B).

An increase in diastolic calcium can induce hypertrophy via activation of the Ca^2+^-dependent CaMKII enzyme. The role of CaMKII signalling in UII-induced hypertrophy was studied in cultured ventricular myocytes treated with 5μM of the CaMKII inhibitor KN-93 for 30 minutes prior to stimulation with 200nM UII [37]. Inhibition of CaMKII signalling pathway completely blocked the UII-induced hypertrophy after 48 hours from (3.39 ± 0.04, N = 6 hearts; 386 cells) in UII group to (4.14 ± 0.06, N = 6 hearts; 223 cells) in UII+KN-93 group (p<0.0001) (Fig 4C).

At the concentrations used PD184352, SB202190 and KN-93 did not show any obvious signs of toxicity (S1 File).

## Discussion

### UII induces hypertrophy in ventricular myocytes

The involvement of UII in hypertrophic heart failure and the associated molecular mechanisms have not been fully elucidated. Whether the increase in circulating UII in patients with heart failure is a cause or effect of left ventricular hypertrophy (LV-hypertrophy) and heart failure is unclear. We determined whether endogenously applied UII is able to induce hypertrophy in adult ventricular tissue.

A growing body of literature has investigated the role of UII in cardiac tissue. In in-vivo and in-vitro animal studies UII-induced hypertrophy and this included increased cellular protein, cell size, collagen synthesis and enhanced myofibrillar reorganization [13, 30, 53]. In our study induction of a hypertrophic response in ventricular myocytes was monitored by measuring length/width ratio. This ratio significantly decreased after 24 and 48 hours stimulation with UII (acting at UT receptor whose presence was demonstrated by PCR), which is indicative of pathophysiological hypertrophy [54]. Moreover the hypertrophic response was reversed by the UT receptor antagonist SB657510 further confirming receptor engagement. To induce hypertrophy, two isoforms of UII (human and rat) were used. Hypertrophic response induced by hUII was similar to that induced by rUII. This confirms that there was no species related differences and agree with those of other studies suggesting equal activity of both isoforms. Potentially this is due to structure of the biologically active C-terminal ring, which is fully preserved in all isoforms of UII across different species [55]. The data of the present study support previous findings in cultured neonatal myocytes; UII at a concentration of 100nM is capable of inducing maximal hypertrophy without upregulation of the UT receptor [13]. Conversely, Onan and co-workers found that the same concentration of UII (100nM) can induce hypertrophy in cultured neonatal myocytes accompanied by overexpression of the UT receptor [14]. Similarly, Gruson and colleagues reported the same findings in adult rat myocytes when UII was incubated with these cells for 48 hours this was associated with activation of GSK-3 [56].

### Intracellular signalling pathways in cardiac hypertrophy

The UT receptor is a G_αq_-coupled GPCR resulting in generation of diacylglycerol (DAG) and inositol triphosphate (IP_3_). DAG activates protein kinase C (PKC) and MAPK subfamilies [57] and IP_3_ releases Ca^2+^ from intracellular stores. In our study UII stimulation increased phosphorylation of ERK1/2 and p38 MAPKs in ventricular myocytes. ERK1/2 was activated at 5–10 minutes and was sustained for up 15 minutes. The present findings seem to be consistent with other studies in neonatal myocytes where ERK1/2 was activated [13, 14]. Our study extends to adult myocytes and shows PD184352 (ERK1/2 inhibition) sensitivity of the hypertrophic response.

Activation of p38 signalling pathway is associated with different pathological stresses in the heart. Most notably, activated p38 is involved in a broad range of cardiac diseases such as hypertrophy, heart failure and myocardial infarction [58]. A number of independent features are stimulated when the p38 signalling pathway is activated during hypertrophy including an increase in myocyte surface area and sarcomere assembly as well as re-expression of specific cardiac hypertrophic foetal genes [3, 59]. We showed activation of p38 within 15 minutes that remaining elevated for at least 60 minutes. The hypertrophic response was sensitive to the p38 antagonist SB202190. Off-target effects of the SB202190 inhibitor includes inhibition of casein kinase 1 (CK1) in human embryonic kidney 293T (HEK 293T) cells [60] but this is unlikely to be important in our study.

Previous studies have reported CaM kinases and calcineurin are implicated in the development of pathological cardiac hypertrophy in response to hypertrophic agents such as Ang II, ET-1 and catecholamines [61–63]. It has been confirmed that expression and activity of CaMKII are upregulated in both animal and human studies of cardiac hypertrophy and heart failure [64–69]. When activated CaMKII leads to re-expression of several hypertrophic foetal genes encompassing, atrial natriuretic peptide, brain natriuretic peptide, skeletal actin and β-myosin heavy chain [70–73].

Using Western blotting we reported time dependent (out to 90mins) UII induced CaMKII activation; the hypertrophic response being blocked by KN93. Stimulation of neonatal myocytes with UII for 48 hours induced hypertrophy via phosphorylation of CaMKII and its downstream phospholamban (PLB) signalling pathway [37]. CaMKII is required for induction of specific hypertrophic genes in response to UII stimulation [74]. Calcium/calmodulin-dependent protein kinase II has various downstream targets which are phosphorylated by CaM kinase and can promote development of heart failure and vascular disease [75].

CaMKII can be activated directly via elevation of intracellular Ca^2+^-transient concentration or alternatively indirectly via intracellular second messenger cyclic adenosine monophosphate (cAMP). As a result of increased cAMP, a guanine nucleotide exchange protein (EPAC) is directly activated. EPAC then activates either phospholipase C or CaMKII resulting in increased intracellular Ca^2+^-release during cardiac excitation-contraction coupling thus contributing to hypertrophy [76–78].

Selective inhibition of CaMKII with KN-93 was used to establish a role in regulating ventricular myocyte hypertrophy. Inhibition of CaMKII signalling pathway completely blocked UII-induced hypertrophy in ventricular myocytes. Co-incubation of KN-93 and UII with neonatal myocytes has been shown to prevent UII-induced phosphorylation of CaMKII and hypertrophic phenotype response as well as increase of protein [37]. The KN-93 inhibitor wraps around the helical CaM target segment and the binding site becomes free from the catalytic kinase domain [79]. Only few protein kinases are influenced by KN-93. KN-93 has a direct effect on unrelated molecules to CaM kinase family [80]. Some studies have shown off target effects, including L-type Ca^2+^ channels [80], voltage-gated K^+^ cannels [81, 82], IP_3_ receptors [83], and calmodulin [84]. Notably, a minimum time was selected for incubation of all inhibitors used along with low concentrations to retain selectivity. Any off-target effects are not likely to be important in our hypertrophy experiments.

In heart failure, hyperphosphorylation of RyR2 at Ser^2808^ mediated by PKA, resulting from β-adrenergic stimulation, impaired FKBP12.6 binding to the RyR2 channels and the final leak of Ca^2+^ from SR [85, 86]. Recently, CaMKII levels have been shown to be elevated in failing hearts, thereby increased phosphorylation of RyR2 at Ser^2008^ and Ser^2814^ (in some species Ser^2815^) residues [87]. RyR2 are not entirely closed and increase their open probability during diastole when these channels are hyperphosphorylated by CaMKII causing a substantial elimination of SR Ca^2+^, leading to a local restriction of SR Ca^2+^-release events (Ca^2+^ sparks) [88, 89]. Spontaneous diastolic opening of single channels can trigger the opening of neighbouring RyRs clusters through the process calcium induced calcium release (CICR) [90, 91]. In this manner, SR Ca^2+^-content is depleted and eventually initiates diastolic SR Ca^2+^-leak.

An increase in diastolic [Ca^2+^]_i_ that might activate CaMKII (a Ca^2+^-dependent enzyme) leading to hypertrophy. The effect of chronic exposure of ARVMs to UII on Ca^2+^-transients was investigated in the present study. The most interesting finding was that there was an increase in diastolic [Ca^2+^]_i_ after 24 hours treatment compared to control. It is possible that these results are due to SR Ca^2+^-leak resulting from hypertrophy. Surprisingly, an increase in diastolic [Ca^2+^]_i_ was not due to an increase in SR Ca^2+^-leak. All of the signalling molecules/cascades were activated in ventricular myocytes within 2 hours. The chronic effects of UII on diastolic [Ca^2+^]_i_ (after 24 hours), shown by our data, may be secondary to the signalling driven via CaMKII or indeed of the resulting hypertrophy. The measurement was probably late and the effect was not seen due to the hypertrophy resulting from the effect of UII within the first 2 hours. Thus, CaMKII could induce RyR2 stimulation leading to diastolic Ca^2+^-leak. To study RyR2, leak experiments should examine the effects over the acute period between 1 and 2 hours. Whilst SR Ca^2+^-leak was not significantly altered, diastolic [Ca^2+^]_i_ was significantly elevated by chronic UII treatment, by a mechanism we could not discern. The increase in diastolic [Ca^2+^]_i_ may also be secondary to the CaMKII signaling. Hypertrophy has been associated with both an increase and decrease in NCX activity [92]. Moreover hypertrophy has also been linked to a decrease in SERCA activity. A decrease in SERCA activity and a decrease in NCX activity could be a reason for the observed increase in diastolic [Ca^2+^]_i_, it could also be secondary to CaMKII activation.

ERK, p38 and CaMKII are downstream signalling pathways mediated of UII/UT system in primary cultured adult or neonatal myocytes whether these cells are isolated from the whole heart or ventricles of rats or the embryonic cardiomyocyte cell line H9c2 [13, 29, 37, 93–95]. Akt/GSK-3β is an additional UII driven downstream effector pathway in neonatal or adult or prenatal myocytes [53, 56, 94]. The current study demonstrated that UII caused pathophysiological hypertrophy (increase in length/width ratio) in adult ventricular cardiac myocytes in culture and UII triggered signalling pathways previously linked to hypertrophic responses. Known inhibitors of these pathways (ERK1/2, p38 and CaMKII) were able to completely block the hypertrophic response. The novelty of the present work is two fold; broad ventricular cardiac myocyte signalling is linked to hypertrophy and there are no apparent published differences between atrial and ventricular or prenatal myocyte responses to UII stimulation.

## Conclusion

In the current study, the ability of exogenous UII to induce hypertrophy in cultured ventricular myocytes was examined. We show a UII hypertrophic response and activation of ERK1/2, p38 and CaMKII. Diastolic [Ca^2+^]_i_ increased in ventricular myocytes treated with UII for 24 hours but there was no increase in SR Ca^2+^-leak. Our data suggest that increased circulating UII may contribute to the development of left ventricular hypertrophy and inhibition may be of clinical benefit.

## Supporting information

S1 Raw images All images were obtained using BioRad ImageLab software as detailed in methods section of the paper. Images are represented in the order they appear in the main text. Molecular weights are included in these images (biotinylated ladder; #7727, Cell signalling). The red box indicates area chosen in figures.**Negative JNK data:** Representative blots indicating that UII did not phosphorylate JNK in ventricular myocytes. UII was incubated with cultured ventricular myocytes for different time points (5, 10, 15, 30 and 45 minutes), the phosphorylation of JNK was not affected by UII treatment at any time point examined. N = 3 hearts.
(PDF)

S1 File Inhibitor dosing and toxicity. PD184352 (ERK1/2 inhibitor), SB202190 (p38 inhibitor) and KN-93 (CaMKII inhibitor) dosing regimen and assessment of gross toxicity.
(PDF)
