# Supplementary material for: Signalling pathways involved in urotensin II induced ventricular myocyte hypertrophy
Source: PLoS One. 2025 Jan 16;20(1):e0313119. doi: 10.1371/journal.pone.0313119 (PMC11737703; doi:10.1371/journal.pone.0313119)
Supplement: S1 Raw images — Images are represented in the order they appear in the main text. Molecular weights are included in these images (biotinylated ladder; #7727, Cell signalling). The red box indicates area chosen in figures.Negative JNK data: Representative blots indicating that UII did not phosphorylate JNK in ventricular myocytes. UII was incubated with cultured ventricular myocytes for different time points (5, 10, 15, 30 and 45 minutes), the phosphorylation of JNK was not affected by UII treatment at any time point examined. N = 3 hearts. (PDF) [file pone.0313119.s001.pdf]

Western blot raw images

All images were obtained using BioRad ImageLab software as detailed in methods section of paper. Images are represented in the order they appear in the main texts. Molecular weights are included in these images (biotinylated ladder; #7727, Cell signalling). The red box indicates area chosen in figures.

Figure 3A

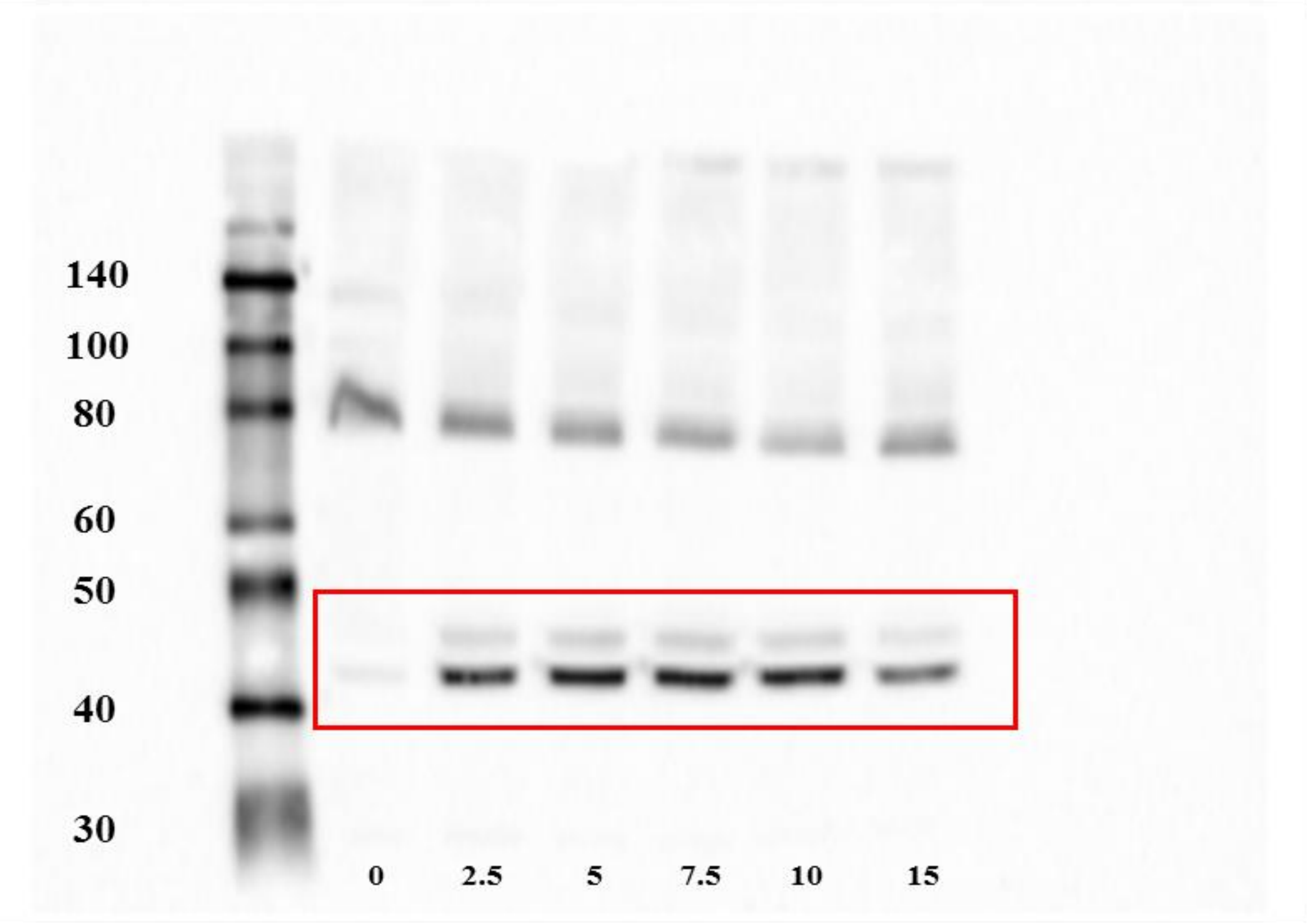

Incubation time (min) of drug

Treated ventricular myocytes with UII phospho-ERK1/2

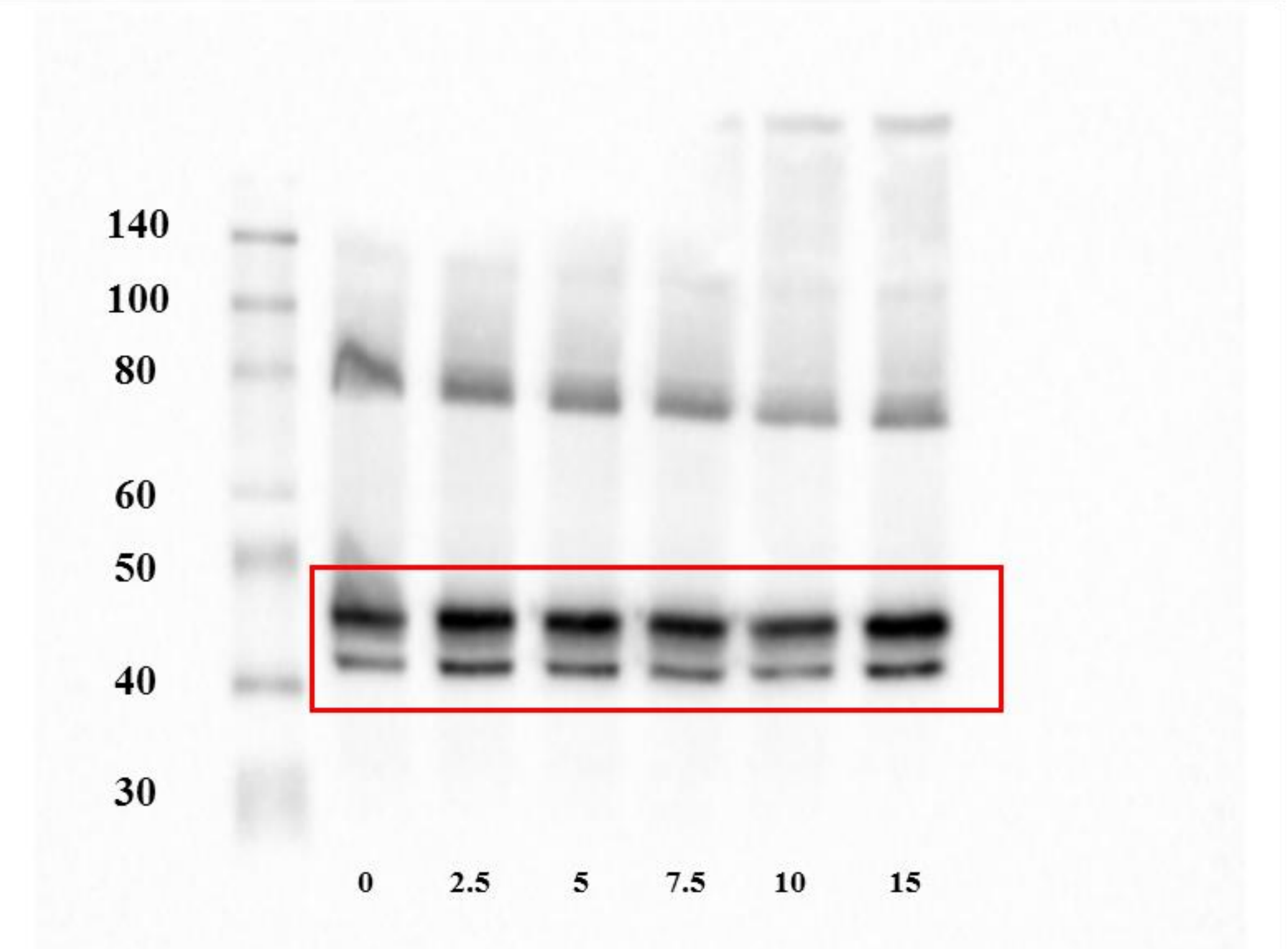

Incubation time (min) of drug

Treated ventricular myocytes with UII total ERK1/2

Figure 3B

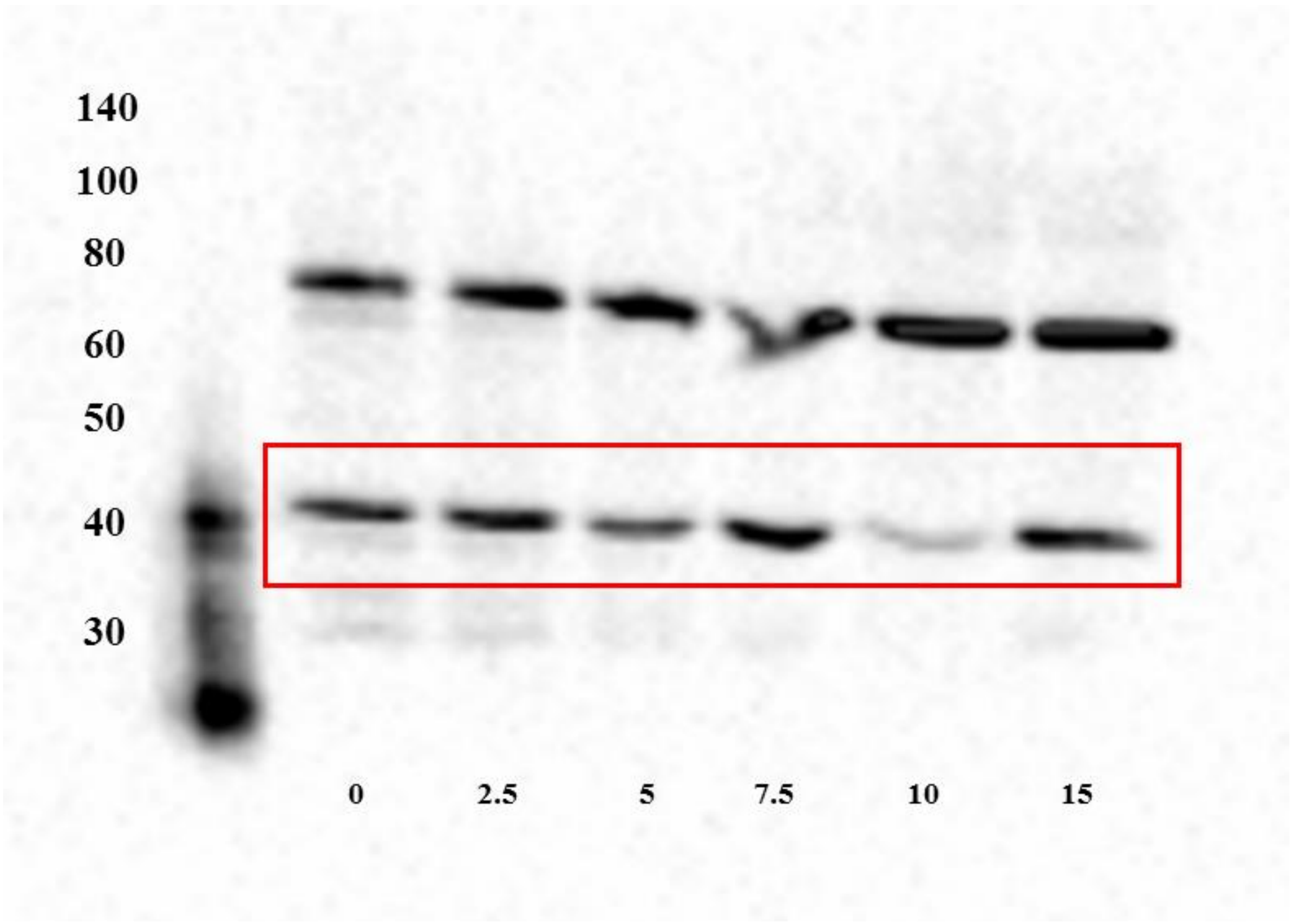

Incubation time (min) of drug

Treated ventricular myocytes with UII phospho-p38

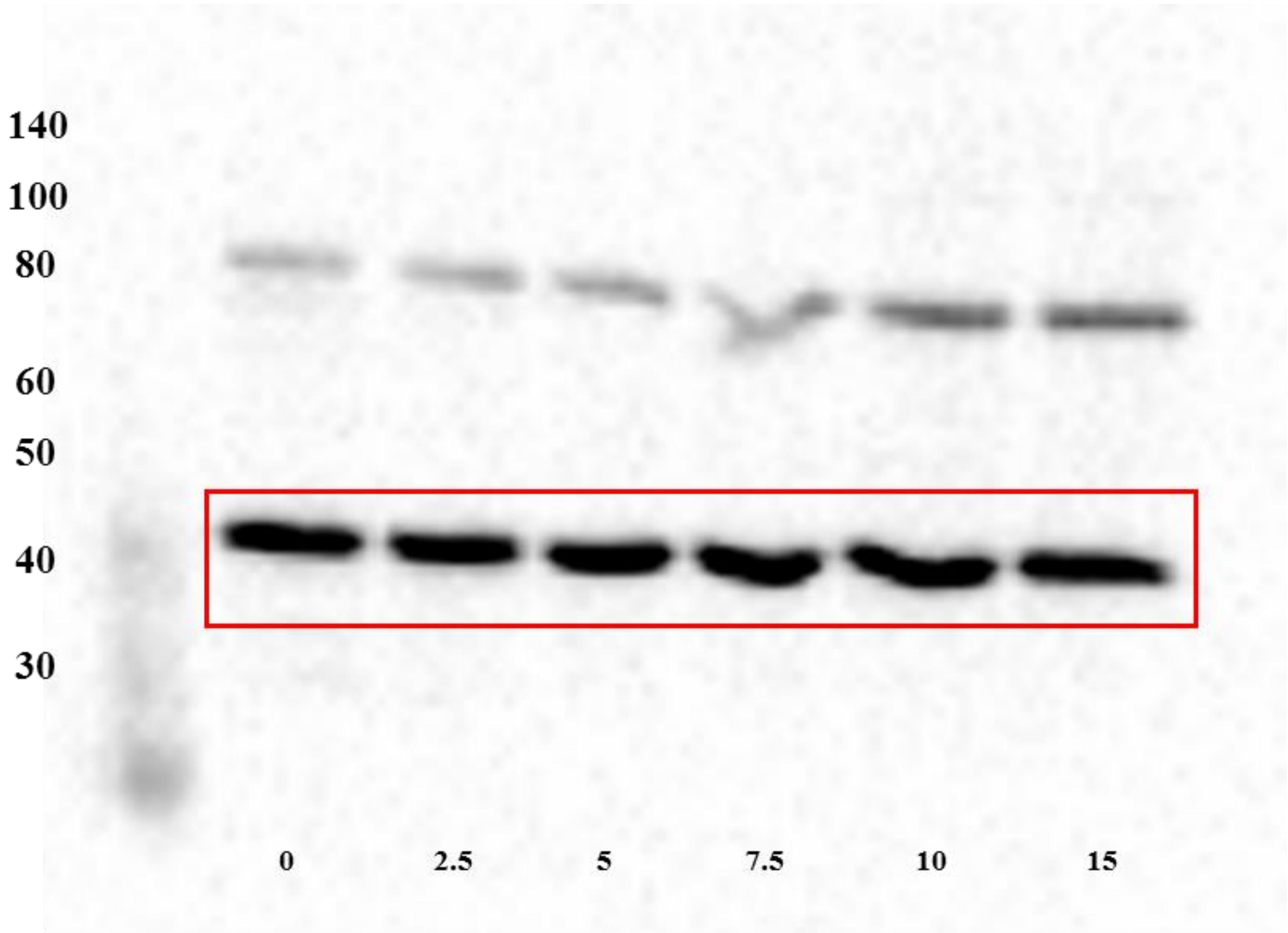

Incubation time (min) of drug

Treated ventricular myocytes with UII total p38

Figure 3B

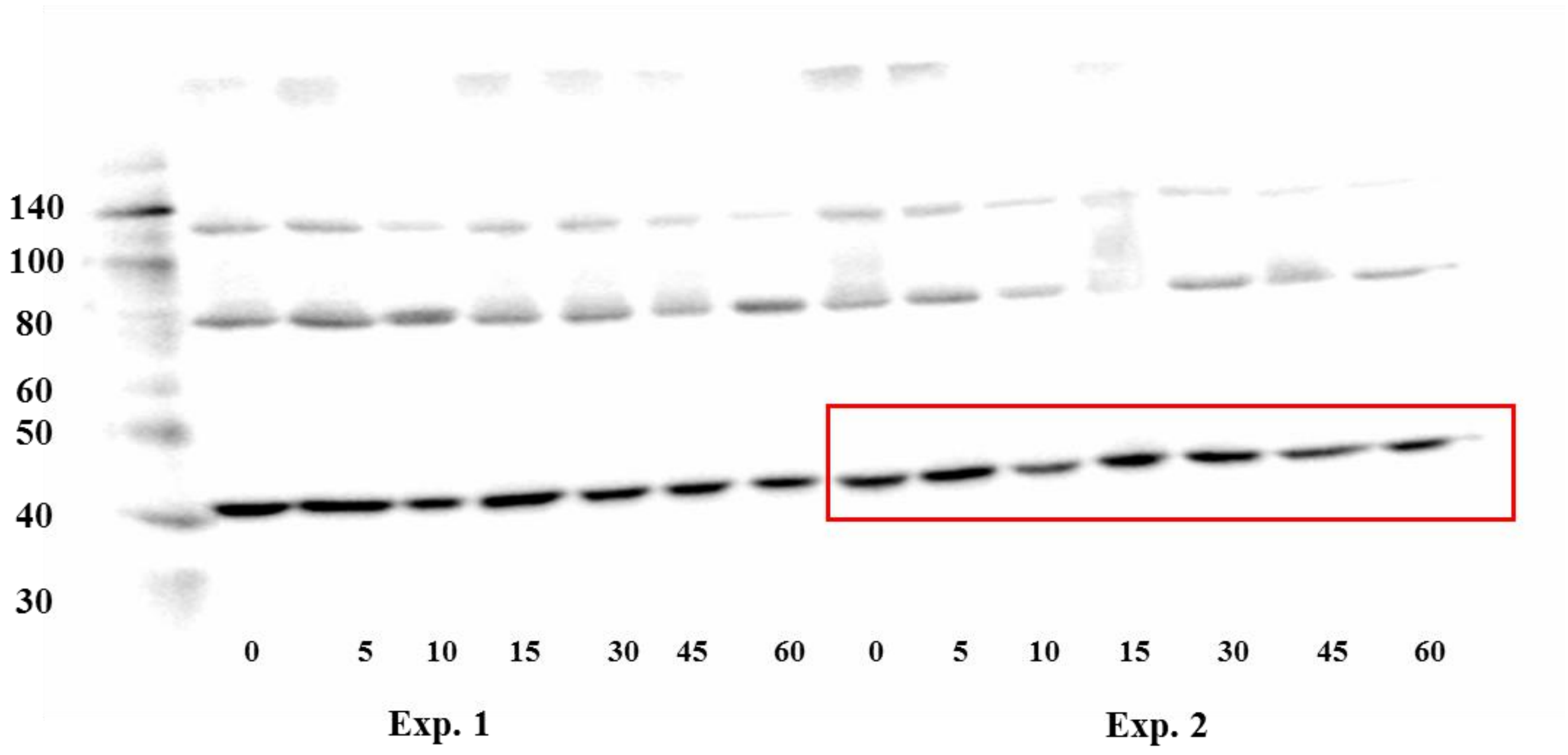

Incubation time (min) of drug

Treated ventricular myocytes with UII phospho-p38

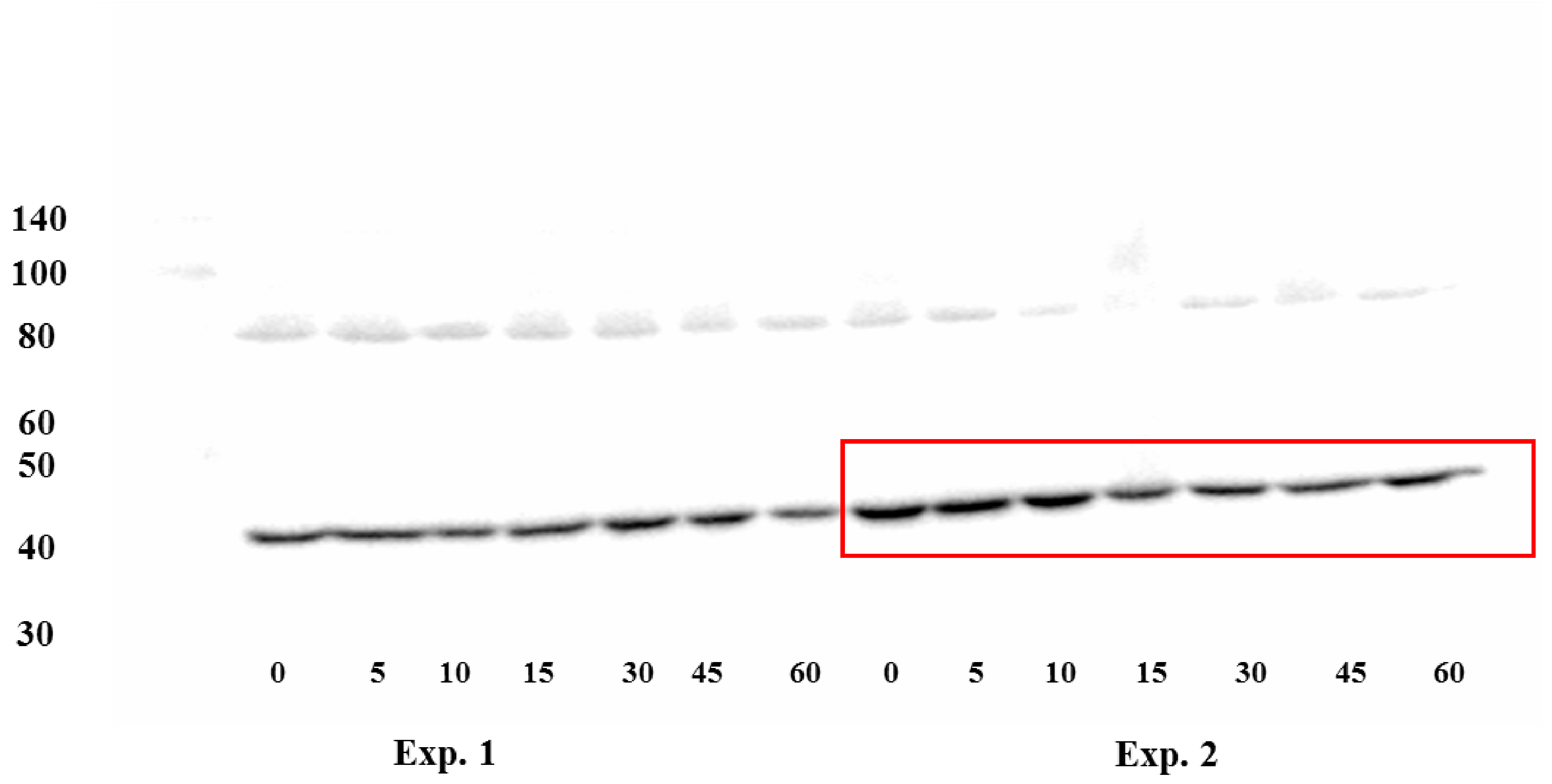

Incubation time (min) of drug

Treated ventricular myocytes with UII total p38

Figure 3C

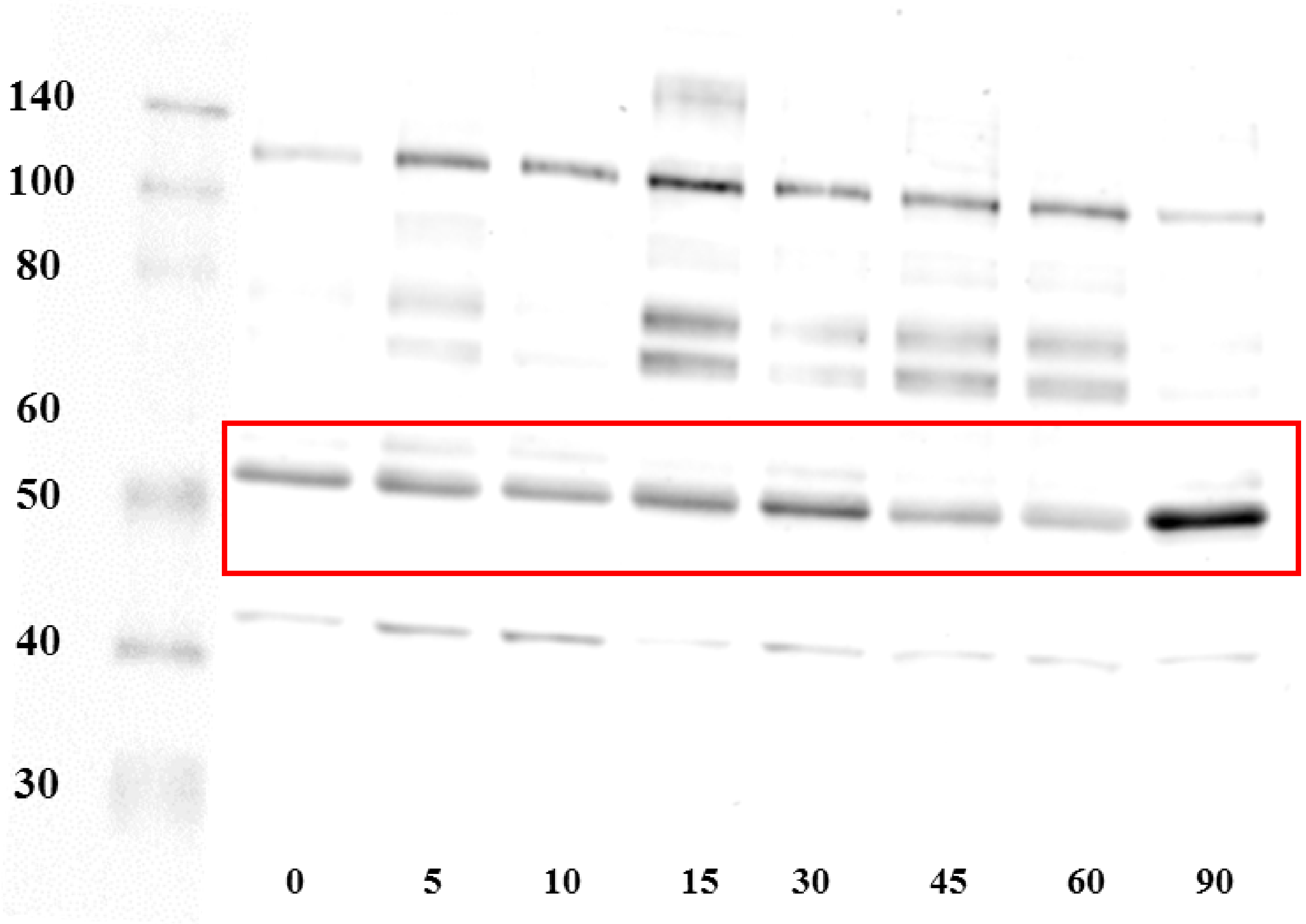

Incubation time (min) of drug

Treated ventricular myocytes with UII phospho-CaMKII

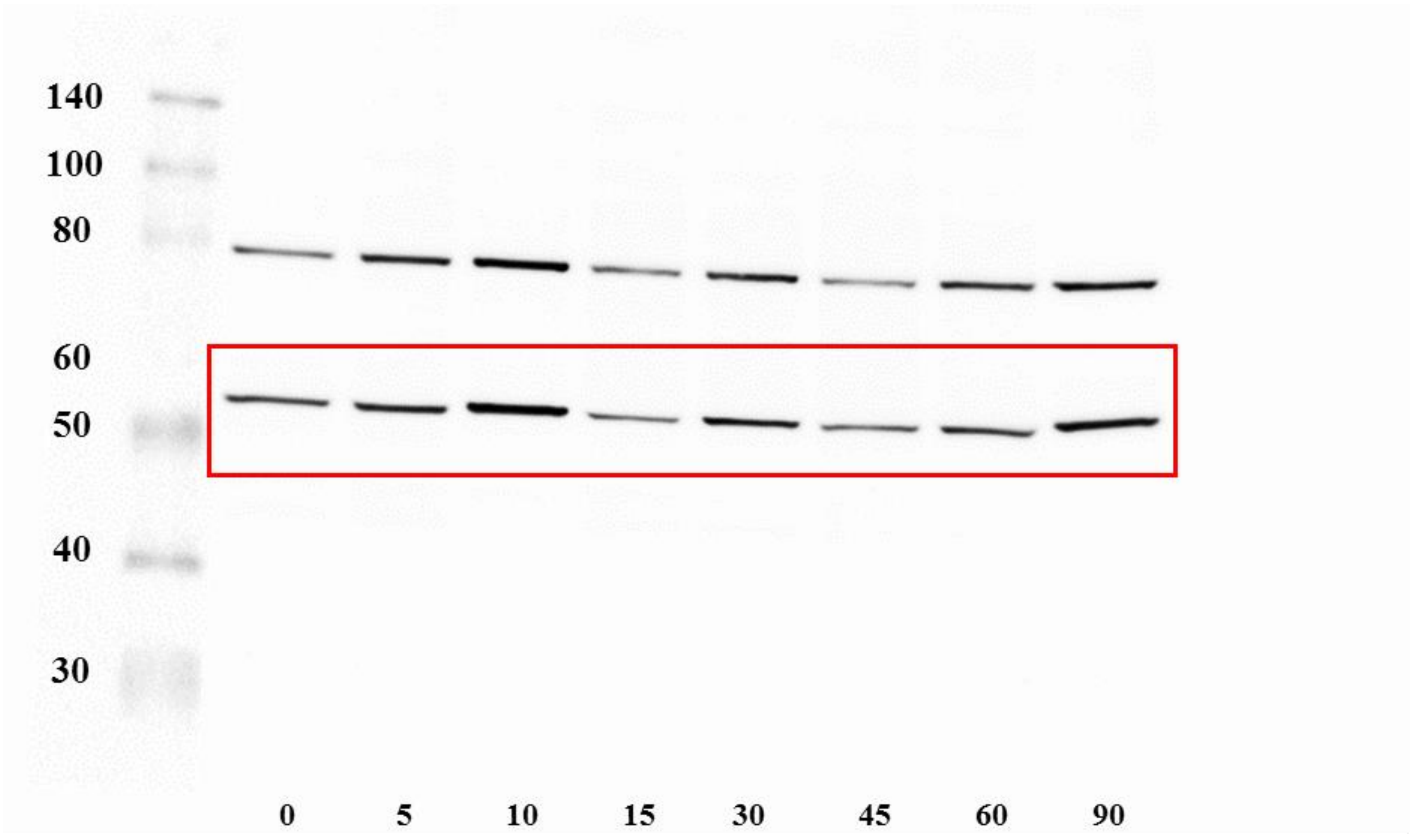

Incubation time (min) of drug

Treated ventricular myocytes with UII total CaMKII

JNK experiment

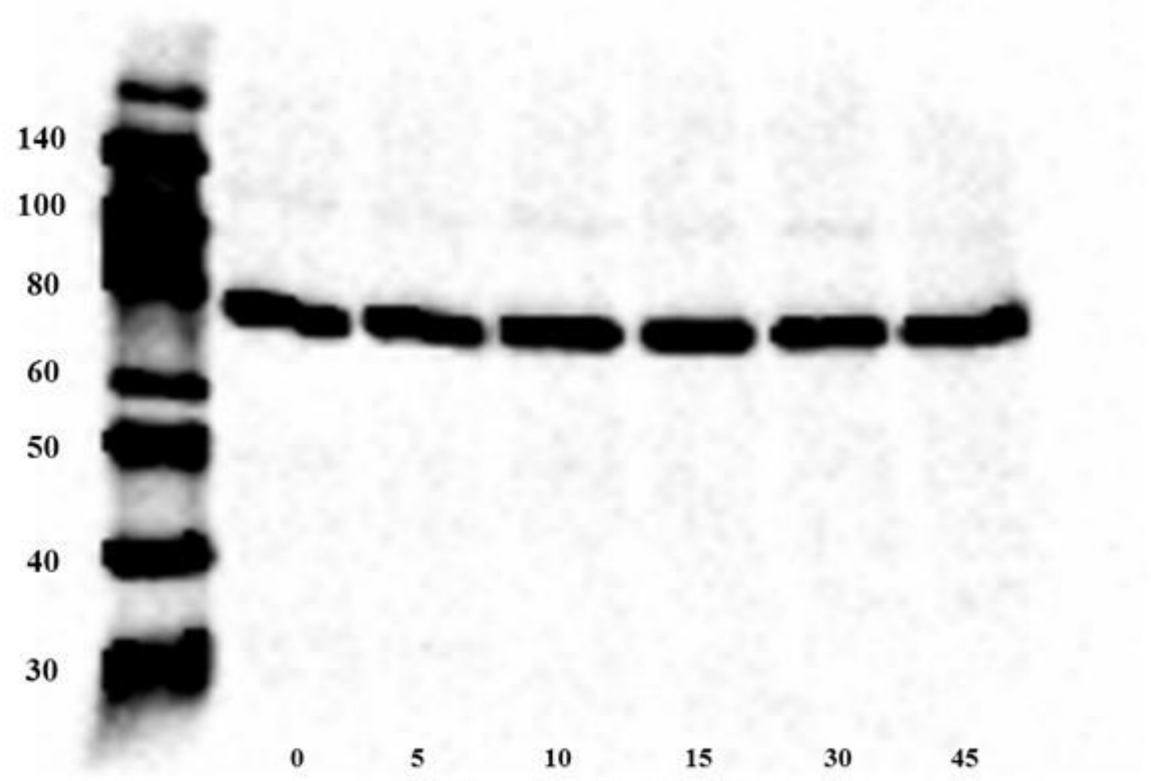

Incubation time (min) of drug

Treated ventricular myocytes with UII phospho-JNK

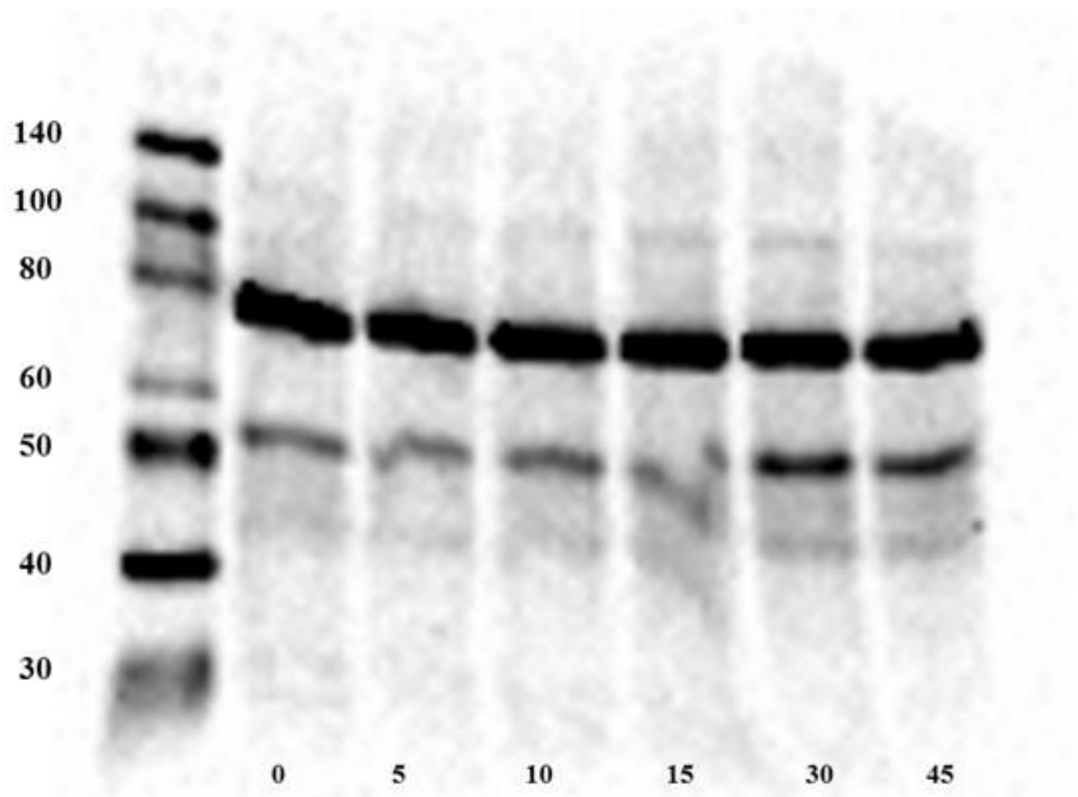

Incubation time (min) of drug

Treated ventricular myocytes with UII total JNK
