# Supplementary material for: Signalling pathways involved in urotensin II induced ventricular myocyte hypertrophy
Source: PLoS One. 2025 Jan 16;20(1):e0313119. doi: 10.1371/journal.pone.0313119 (PMC11737703; doi:10.1371/journal.pone.0313119)
Supplement: S1 File — PD184352 (ERK1/2 inhibitor), SB202190 (p38 inhibitor) and KN-93 (CaMKII inhibitor) dosing regimen and assessment of gross toxicity. (PDF) [file pone.0313119.s002.pdf]

Supplement-2: PD184352 (ERK1/2 inhibitor), SB202190 (p38 inhibitor) and KN-93 (CaMKII inhibitor) dosing regimen and assessment of gross toxicity.

A. Inhibitor dosing.

**ERK1/2 inhibitor (PD184352)**		
Authors	Dose	DOI
Ley et al. 2003	5µM	10.1074/jbc.M301010200
Yu et al. 2015	5µM	10.1371/journal.pone.0144433
Smith et al. 2012	5µM	10.1093/jnci/djs471
**P38 inhibitor (SB202190)**		
Authors	Dose	DOI
Chen et al. 2012	10µM	10.12659/MSM.883477
Schwartz et al. 2018	10µM	10.18632%2Foncotarget.25234
Yang et al. 2020	10µM	10.1016%2Fj.redox.2020.101445
Karahashi et al. 2000	10µM	10.1016/S0925-4439(00)00045-4
Papanicolaou et al. 2023	10µM	10.1016%2Fj.jbc.2023.102907
Meng et al. 2021	10µM	10.1038/s41419-021-04395-z
**CaMKII inhibitor (KN-93)**		
Authors	Dose	DOI
Shi et al. 2010	5µM	10.1016/j.gene.2016.02.039
Cipolletta et al. 2015	5µM	10.1371/journal.pone.0130477
Li et al. 2023	5µM	10.1016/j.mad.2022.111758
Alves-Figueiredo et al. 2024	5µM	10.1016/j.jacbts.2024.01.007

B. Effect of inhibitors on Length/Width (LW) ratio.

Effects of ERK1/2 (PD184353, 5µM) and p38 (SB202190, 10µM) inhibitors

	**1h**	**24h**	**48h**
Control	4.80±0.07 (328)	4.58±0.05 (528)	4.87±0.07 (358)
+PD184353	4.81±0.08 (233) ^{NS}	4.96±0.07 (330) *	4.80±0.07 (253) ^{NS}
+SB202190	4.83±0.09 (256) ^{NS}	4.64±0.06 (346) ^{NS}	4.80±0.07 (278) ^{NS}

Effects of CaMKII (KN-93, 5µM) inhibitor

	**1h**	**24h**	**48h**
Control	4.10±0.06 (328)	3.96±0.05 (319)	4.08±0.06 (290)
+KN-93	4.10±0.06 (305) ^{NS}	4.06±0.06 (257) ^{NS}	4.22±0.07 (282) ^{NS}

Effect of ERK1/2 inhibitor (PD184352), p38 inhibitor (SB202190) and CaMKII inhibitor (KN-93) on resting (unstimulated) LW ratio in cultured ventricular myocytes. LW ratio data are expressed as mean±S.E.M. With the exception of a small (7.6%) but statistically significant (One-way ANOVA followed by Sidak's *post hoc* test) effect of PD184353 at 24h only there were no effects of the inhibitors alone.
